# Editorial for Special Issue: “Recent Advances in Green Solvents”

**DOI:** 10.3390/molecules28165983

**Published:** 2023-08-10

**Authors:** Reza Haghbakhsh, Sona Raeissi, Rita Craveiro

**Affiliations:** 1 Department of Chemical Engineering, Faculty of Engineering, University of Isfahan, Isfahan 81746-73441, Iran; 2 LAQV, REQUIMTE, Departamento de Química da Faculdade de Ciências e Tecnologia, Universidade Nova de Lisboa, 2829-516 Caparica, Portugal; rita.craveiro@campus.fct.unl.pt; 3 School of Chemical and Petroleum Engineering, Shiraz University, Shiraz 71345-51154, Iran; raeissi@shirazu.ac.ir

## 1. Introduction

Today, environmental conservation is one of the most urgent targets. Fortunately, this goal is taken into consideration in the general policies of most countries. Accordingly, worldwide development needs to be aligned with environmental considerations [1]. In all countries, the industrial sector inevitably has the most impact on sustainable development. Green chemistry, by considering environmental issues affecting the planet and its living creatures, provides useful guidelines for any kind of sustainable development, especially in the industries. This can be achieved by screening and recommending novel ideas, methods, processes, etc. In this way, green chemistry can be considered as a general scientific field, with 12 principles. The fifth principle of green chemistry is “Safer Solvents and Auxiliaries”, which directly emphasizes the importance of green and environmentally friendly solvents [2]. Following this principle, in the past few decades, the general idea of replacing conventional hazardous and harmful solvents with green solvents has been highlighted in scientific communities and research centers in industries. Various types of green solvents, such as supercritical fluids, ionic liquids (ILs), deep eutectic solvents (DESs), etc., have shown high levels of potential in many applications. The number of published studies on the topic of green solvents has significantly increased year on year [3]. Therefore, “green solvents” can be considered a hot topic of green chemistry, deserving more specific investigations in scientific publications. Because of this, *Molecules* has devoted a Special Issue to recent advancements in the interdisciplinary area of green solvents. In this Special Issue, fundamental as well as application-based studies and innovative techniques regarding green solvents were covered. We were delighted to welcome interesting, high-quality, and valuable studies in this field.

## 2. Contributions

This Special Issue includes eleven original research articles covering various aspects of green solvents, including fundamental knowledge and applications.

Huang et al. [4] studied the performance of ionic liquids for the extraction of heavy metals. They experimentally investigated the efficiency of 1-butyl-3-methylimidazolium chloride in extracting Cr(VI) from a Cr-contaminated simulated sorbent in soil remediation.

Kostenko and Parenago [5] considered the application of supercritical CO_2_ for impregnation in place of organic solvents, in order to prepare sorbents based on hyper crosslinked polystyrene and the chelating agent N,N,N′,N′-tetraoctyl diglycolamide. They proposed an environmentally friendly and green solvent for this process.

Nowosielski et al. [6] carried out fundamental research by investigating important physical properties (density, speed of sound, refractive index, and viscosity) of both pure and aqueous solutions of a number of deep eutectic solvents. They studied DESs made of tetrabutylammonium chloride + 3-amino-1-propanol and tetrabutylammonium bromide + 3-amino-1-propanol or 2-(methylamino)ethanol or 2-(butylamino)ethanol.

As a comprehensive study, Zailani et al. [7] reported the results of their research on the synthesis of a series of ammonium cations coupled with carboxylate anions producing ammonium-based protic ionic liquids, and their densities, viscosities, refractive indices, thermal decomposition temperatures, glass temperatures, and CO_2_ absorption was also reported.

In an application-based study, da Silva et al. [8] proposed the use of supercritical CO_2_ to extract non-volatile compounds from *A. mearnsii* flowers. They concluded that the extracted essence showed antimicrobial activity. They also showed the presence of p-anisic acid, a substance with industrial and pharmaceutical applications.

Also, in the field of energy, Peyrovedin et al. [9] showed that green solvents can be useful in solar energy plants, and in addition to showing how they make the process eco-friendly, they also elaborated on its good performance. They studied the feasibility of using a number of deep eutectic solvents as phase change material (PCM) for solar thermal power plants with organic Rankine cycles.

In another article, Chinchilla et al. [10] presented applications of green solvents in the field of catalysts. They proposed high-temperature water reactions to reduce CO_2_ by using an organic reductant and a series of metals and metal oxides as catalysts.

Ghigo et al. [11] studied deep-eutectic-solvent-like mixtures, based on glycerol and different halide organic and inorganic salts, as new high-potential media in the copper-free halodediazoniation of arenediazonium salts. They reported the experimental results of the reaction and also presented a computational investigation to understand the reaction mechanism.

Lee et al. [12] investigated the applications of deep eutectic solvents in extractions from natural leaves. They compared the efficiencies of various ratios of choline chloride and dicarboxylic acids for the extraction of flavonoid components from *Pyrus ussuriensis* leaves with respect to conventional solvents. 

In a fundamental research article, Ma et al. [13] studied the atomic structure of cellulose that was dissolved in alkali/urea aqueous solutions. They used trehalose as the model molecule with total scattering as the main tool to study three kinds of alkali solution, consisting of LiOH, NaOH, and KOH. They reported interesting findings on the most probable all-atom structures of the solution, the hydration shell of trehalose, the penetration of ions into glucose rings, and the molecule interactions of urea with hydroxide groups.

Panić et al. [14], by proposing a new modeling strategy, presented their experimental and modeling investigations into the development of a simple and straightforward model to estimate the pH values of deep eutectic solvents over a wide range of values. They developed the model according to a large number of deep eutectic solvents, using artificial intelligence techniques for modeling.

In the last article in this Special Issue, Osman et al. [15] studied the application of a new green solvent for biodiesel purification via Solvent-Aided Crystallization (SAC). Biodiesel purification is an important experimental step in biodiesel synthesis, and it would be more beneficial to consider green solvents for this task. In their work, they also investigated the technological improvements in the purification of biodiesel via SAC and compared the performance of a new green solvent with conventional solvents in the production of high-purity biodiesel.

## 3. Conclusions

The concept of green solvents is not new, and supercritical fluids and ionic liquids have led the way in this field. Green solvents are dynamic and continuously thriving, with new solvents and new categories of solvents being added over time. Deep eutectic solvents, as a recently introduced category of green solvents, are highly significant and may encompass huge numbers of members. Currently, new ILs and DESs are being introducing to the scientific community at a rapid pace. Consequently, wide ranges of investigations, from fundamental research for new members to application-based research for well-known members, are all vital and important from their own perspectives. One of the aims of this Special Issue was to highlight the wide scope of research being carried out on green solvents and to involve a variety of researcher and reader orientations. We have included a range of studies, from fundamental research on physical properties and reaction kinetics to application-based studies such as essence extraction, impregnation via supercritical fluids, and biodiesel purification. We have also covered the even more specific field of energy for solar power plants using green solvents.

## Data Availability

Not applicable.
